# Transcriptome and digital gene expression analysis unravels the novel mechanism of early flowering in *Angelica sinensis*

**DOI:** 10.1038/s41598-019-46414-2

**Published:** 2019-07-11

**Authors:** Guang Yu, Yuan Zhou, Juanjuan Yu, Xueqin Hu, Ye Tang, Hui Yan, Jinao Duan

**Affiliations:** 1 School of Medicine and Life Sciences, Nanjing University of Chinese Medicine, Nanjing, China; 2 Jiangsu Collaborative Innovation Center of Chinese Medicinal Resources Industrialization, Nanjing University of Chinese Medicine, Nanjing, China

**Keywords:** Plant sciences, Plant molecular biology, Plant sciences, Plant molecular biology

## Abstract

*Angelica sinensis* (Oliv.) Diels is a widely used medicinal plant mainly originated in Gansu, China. *Angelica sinensis* is greatly demanded in the clinical practice of Chinese medicine due to its broad pharmacological activities of hematopoietic and anti-inflammatory properties. But, the percentage of early flowering in *Angelica sinensis* arrives to 20%~30%, which severely affects its quality and quantity. Here, transcriptome profiling and digital gene expression analysis were applied to study the mechanism of early flowering in *Angelica sinensis*. A total of 49,183,534 clean reads were obtained and assembled into 68,262 unigenes, and 49,477 unigenes (72.5%) could be annotated to a minimum of one database in the Nr, Nt, Swiss-Pro, GO, COG and KEGG. Taking the above transcriptome data as a reference, digital gene expression result showed that 5,094 genes expression level were significant changed during early flowering. These annotated genes offered much information promoting that the biosynthesis of secondary metabolites pathway, the hormone signal transduction pathway, and the transcription regulation system may be closely related to the early flowering phenomenon of *Angelica sinensis*. Further expression patterns of key genes contribute to early flowering were analyzed using quantitative real-time PCR. The transcriptome result offered important gene expression information about early flowering in *Angelica sinensis*.

## Introduction

*Angelica sinensis* (Oliv.) Diels (*AS*) is a world-renowned plant medicine originating in Gansu, China. The root of *AS* is traditional Chinese medicinal materials, which was firstly recorded in “*Shen Nong Ben Cao Jing*” in the Han Dynasty. *AS* is suggested as a tonic, hematopoietic, antitumor^1,2^, and anti-inflammatory^3,4^ properties for the treatment of menstrual disorders, amenorrhea, dysmenorrhea. It is also considered as a healthy food material in Asia, Europe and America^5^. Thus, *AS* is great required in the world due to its widely used.

However, the early flowering rate of *AS* reaches to 20%~30%, once the early flowering occurs, the roots of *AS* will be lignified and cannot be used as medicine, which severely affects its quality and quantity. Previous researches were focused on the function of ecological and nutritional factors in affecting early flowering of *AS*. However, the molecular mechanism of early flowering of *AS* is still elusive. To date, there is no effective methods to control and prevent the early flowering of *AS*. The molecular mechanism of early flowering in *AS* needs to be further studied.

The transcriptome assembly provided a powerful tool for high-throughput research at the genetic level^6^. Digital gene expression (DGE) analysis was also considered as a valuable tool to quantitative comparison gene expression, which can efficiently screen and comment differentially expressed genes^7^. In non-model plants, combine these two methods can promote the identification of different expression genes. The combination of transcriptome sequencing and DGE can provide more sensitive and efficient analysis of gene expression changes, and can also promote gene expression comparisons for the sample without reference databases^8^.

Here, Illumina sequencing technology and DGE system were performed to study the gene expression changes in early flowering of *AS*. DGE libraries from apical meristem of vegetative growth *AS* and flower buds of early flowering *AS* were built. By comparing changes in gene expression between these different groups, we can understand more deeply in the molecular mechanism of early flowering in *AS*.

## Material and Methods

### Plant materials

*AS* is a triennial medicinal plant, which flowers in the third year. Early flowering is frequent in May of the second year. The flower buds of early flowering *AS* and apical meristem of vegetative growth *AS* were collected in June of the second year in the field of Minxian (located at 34°29′ North latitude and 103°57′ East longitude), Gansu Province, China. The samples were stored at −80 °C. The medicinal plant was identified by Hui Yan, associate professor of Pharmacognosy, Nanjing University of Chinese Medicine.

### RNA preparation and cDNA synthesis

Total RNA from the plant materials was extracted and identified through a TRIzol/chloroform (Life Technologies, Carlsbad, California, USA) referenced to the manufacture’s protocols.

### Transcriptome library preparation and sequencing of *AS*

The RNA was extracted from the flower buds of early flowering *AS* and apical meristem of vegetative growth *AS* for transcriptome analysis. If the RIN ≥8 and a 260/280 nm absorption ratio ≥ 1.8, RNA was used to set up transcriptome library. After the RNA extraction, mRNA was purified from total RNA by binding the RNA to magnetic beads. Then, mRNA was broken into short fragments. The cleaved RNA fragments were used as templates to synthesize the first-strand cDNA, after that DNA polymerase I and RNase H were added to synthesize the second-strand cDNA.

Next, suitable fragments were used as templates for PCR amplification, which yielded as the cDNA library for sequencing.

### *De novo* assembly and unigenes annotation

The clean reads were screened from the raw data by filtering out poly-N, the low-quality reads (quality value ≤ 10 or reads including more than 5% unknown nucleotides). Then, the unigenes were generated by *De novo* assembling of the clean reads by using Trinity method^9,10^. In order to understand the function of the unigenes, they were searched against the public databases, including NCBI Nr and Nt, Swiss-Prot, GO, COG, and KEGG database, with *E* value ≤ 10^−5^.

### Digital gene expression library preparation and sequencing

DGE library preparation of the three groups of *AS* Samples were performed in parallel using an Illumina Gene Expression Sample Preparation Kit (ZC: apical meristem of vegetative growth *AS*, ZT1:early stage flower bud of early flowering *AS* (During the *AS* early flowering time window, we observed the *AS* plants in the field every day, and the flower buds was collected within three days, the length of flower bud is less than 5 mm normally) and ZT2: late stage flower bud of early flowering *AS* (the flower buds was collected within one week, the length of flower bud is less than 1 cm normally)). Each experimental group consists of three biological sample replicates (no technical replicates).

### Identification of differentially expressed genes

The clean reads from the sample of apical meristem of vegetative growth *AS* and flower bud of early flowering *AS* were mapped with the transcriptome library above. Reads per kilobase of per million mapped reads (RPKM) was used to measure the gene expression level. If the genes are satisfied with two conditions, false discovery rate (FDR) ≤ 0.001 and an absolute value of log2Ratio ≥ 1, they were defined for significant expression differences. The different expression genes (DEGs) were then compared with the transcriptome library of *AS* above.

### Quantitative real-time PCR analysis

In order to verify the reliability of the DGE results, qRT-PCR was applied using LightCycler 480 SYBR Green I Master Mix (Roche, Basel, Switzerland) and a LightCycler 480 II Real-Time PCR instrument (Roche, Basel, Switzerland). Briefly, 1 μL of cDNA template from different group was used for reaction. The result of each gene repeated at least 3 times. The candidate genes expression changes were analyzed using 2^−△△CT^ method. Glyceraldehyde-3-phosphate dehydrogenase (GAPDH) was used as the endogenous control.

## Results

### Assembly of transcriptome sequencing

**Figure 1:**
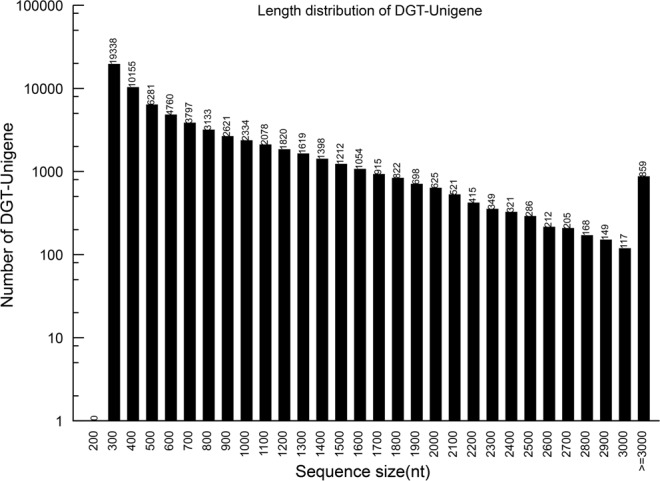
Length distribution of the unigenes from the sample.

**Figure 2:**
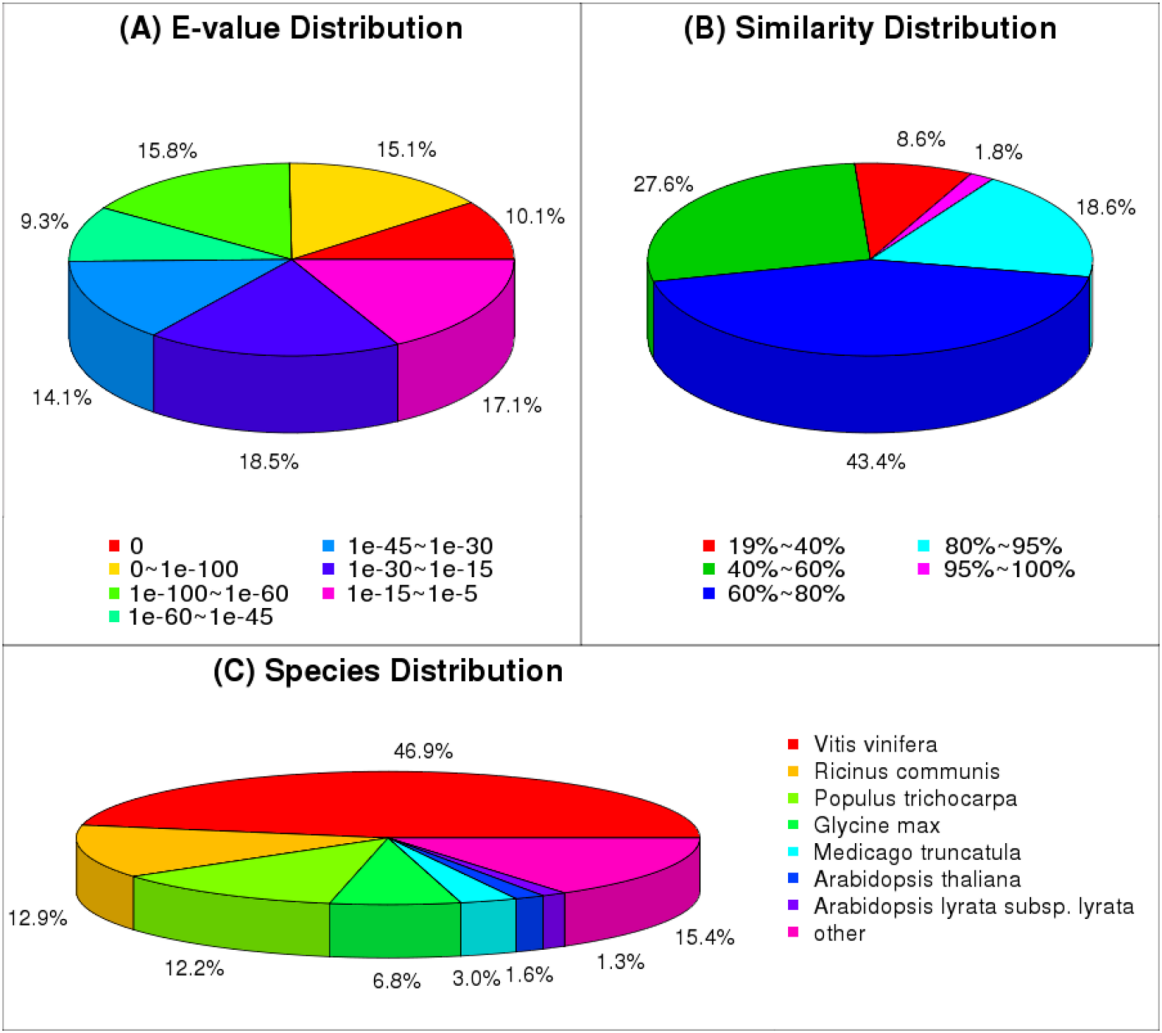
Data of NR classification. (**A**) E-value distribution of BLAST hits for each unique sequence. (**B**) Similarity distribution of the top BLAST hits for each sequence. (**C**) The species distribution is shown as a percentage of the total homologous sequences.

Because the genome sequencing of *AS* has not yet been carried out, it is necessary to complete the sequencing of transcriptome of *AS* to provide a reference for screening differential expression of genes during early flowering. After filtering the adaptors and low-quality sequences, there are 49,183,534 clean reads (Table 1). In addition, the GC percentage is 44.41% and the Q20 percentage is 98.18% of *AS* library. Subsequently, 133,010 contigs were assembled by short reads with average lengths of 90 bp (Table 2). Then a total of 68,262 unigenes were assembled (including 25,560 clusters and 42,702 singletons) by Trinity with a average length of 728 bp (Fig. 1, Table 2). The E-value and similarity distribution against the NR database were showed in Fig. 2A,B. All raw data is public visible (biosample accession number: SAMNO6335422).

**Table 1:** Summary of the *Angelica sinensis* (Oliv.) Diels transcription.

Sample	Total raw reads	Total clean reads	Total clean nucleotides (nt)	Q20 percentage	N percentage	GC percentage
AST	52,786,506	49,183,534	4,426,518,060	98.18%	0.00%	44.41%

**Table 2:** Statistics of assembly quality.

	Sample	Total number	Total length (nt)	Mean Length (nt)	N50	Total Consensus Sequences	Distinct Clusters	Distinct Singletons
Contig	AST	133,010	45,498,302	342	587			
Unigene	AST	68,262	49,709,526	728	1087	68,262	25,560	42,702

### Annotation of *AS* transcripts

In order to understand the comprehensive function of the unigenes, the 68,262 unigenes were searched against the NT, NR, Swiss-Prot, GO, COG and KEGG databases. Search results indicated that 49,477 unigenes had homologous sequences, including 48,106 in NT, 37,408 in NR, 29,840 in Swiss-Prot, 26,438 in KEGG, 15,688 in COG, and 38,205 in GO (Table 3). In terms of distributed species, the homologous genes matched with the unique sequences of *AS* were mainly concentrated in *Vitis vinifera* (46.9%), followed by *Ricinus communis* (12.9%), *Populus trichocarpa* (12.2%) (Fig. 2C).

**Table 3:** Statistical results of unigene annotations.

Database type	NT	NR	SwissProt	KEGG	COG	GO	Total Unigenes
Number of unigenes	48,106	37,408	29,840	26,438	15,688	38,205	49,477
Percentage (%)	97.2	75.6	60.3	53.4	31.7	77.2	

### Classification of *AS* transcripts

**Figure 3:**
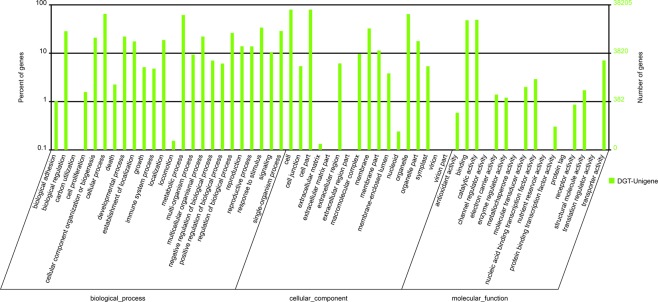
Gene ontology (GO) classification of AS transcriptome. Results are summarized for three main GO categories: biological process, cellular component, molecular function. The right and left y-axis indicate the number and the percentage of each GO term respectively.

Using WEGO software program, 326,207 unigenes were categorized in three categories: biological process, cellular component, and molecular function (Fig. 3). Because some unigenes matched to a few function groups, the number of unigenes match to the biological process was 161,138, to the cellular component was 120,997, to the molecular function was 44,072. In molecular function category, 18,759 unigenes were assigned to “catalytic activity” and 18,353 unigenes were assigned to “binding”, which are the largest proportion, including 84.21% of the total unigenes. In cellular component category, “cell” (30,491), “cellpart” (30,491) and “organelle” (24,576) were highly represented. Moreover, “cellular process” (24,859) and “metabolic processes” (23,613) were the main groups in biological process category.

**Figure 4:**
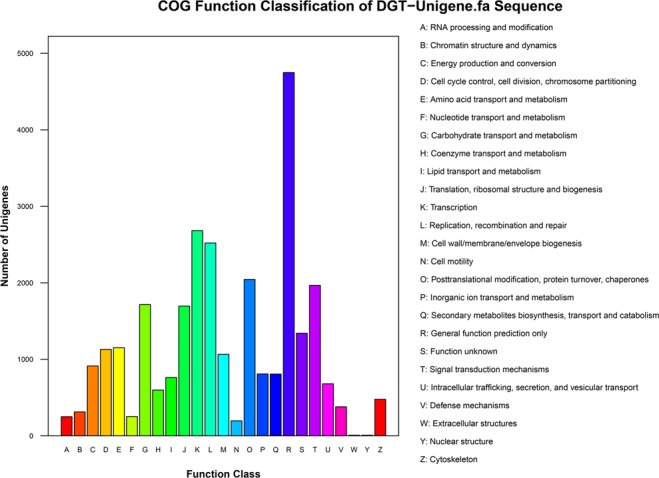
Histogram of COG classification. In total, 68,262 unigenes were classified into 25 categories.

Clusters of Orthologous Group as a database was used for functional prediction and classification of unigenes. By searching with the COG database, 28,513 unigenes were assigned to 25 categories based on COG functional classification (Fig. 4). The number (4,749) of unigenes matching “General functional prediction only” was the highest in all category, followed by “Transcription” (2,682), “Replication, recombination and repair” (2,521), “Post-translational modification, protein turnover, chaperones” (2,044) and “Signal transduction mechanisms” (1,967). The number of unigenes matching “Nuclear structure” (7) and “Extracellular structures” (7) were the least.

**Figure 5:**
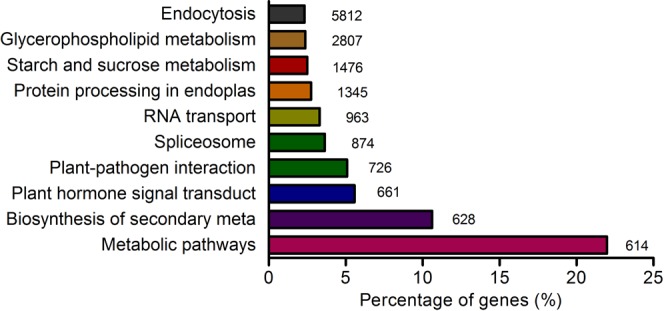
The top 10 annotated unigene pathways.

To further discovery the biological pathways that involved in the early flowering of *AS*, the unigenes were mapped with the pathways in the KEGG database. The results showed that 26,438 unigenes were mapped to 128 predicted metabolic pathways (Fig. 5). The largest category was “Metabolism pathway” (5,812), “Biosynthesis of secondary metabolites” (2,807), “Plant hormone signal transduction” (1,476), “Plant-pathogen interaction” (1,345), “Spliceosome” (963), “RNA transport” (874), “Protein processing in endoplasmic reticulum” (726), “Starch and sucrose metabolism” (661), “Glycerophospholipid metabolism” (619), and “Endocytosis” (614).

### Digital gene expression library sequencing and mapping

**Figure 6:**
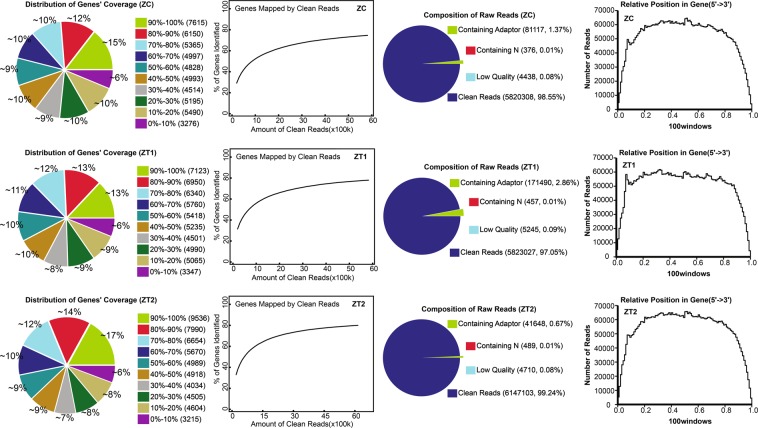
The qualify of transcriptome sequencing was measured by distribution, saturation, homogenization and randomness.

The gene expression changes involved in the early flowering of *AS* were identified by DGE analysis. The sequencing saturation, homogenization and randomness were used reflect the quality of sequencing, and decided whether the data are suitable for further gene expression difference analysis. The distribution of a gene’s coverage was considered as one of the most important parameter to measure the quality of the DGE libraries sequence dataset. In our results, the coverage of 56% of unigenes was exceeded 50% in all DGE libraries (Fig. 6).

### Read mapping

The differentially expressed genes (DEGs) between samples were identified using an algorithm. The matching percentage of clean reads and reference genes ranged from 87.47% and 88.90% in three DGE libaary. Among all reads, 67.50–69.74% per library was uniquely mapped to the reference genome, and 78.62–80.20% of reads was a perfect match to the reference gene (Table 4).

**Table 4:** Summary of read mapping.

Sample ID	Total Reads	Total BasePairs	Total Mapped Reads	Perfect Match	< = 2 bp Mismatch	Unique Match	Multi-position Match	Total Unmapped Reads
ZC	5,820,308 (100.00%)	285,195,092 (100.00%)	5,174,425 (88.90%)	4,667,875 (80.20%)	506,550 (8.70%)	4,059,077 (69.74%)	1,115,348 (19.16%)	645,883 (11.10%)
ZT1	5,823,027 (100.00%)	285,328,323 (100.00%)	5,122,307 (87.97%)	4,612,729 (79.22%)	509,578 (8.75%)	3,990,294 (68.53%)	1,132,013 (19.44%)	700,720 (12.03%)
ZT2	6,147,103 (100.00%)	301,208,047 (100.00%)	5,376,991 (87.47%)	4,832,580 (78.62%)	544,411 (8.86%)	4,149,173 (67.50%)	1,227,818 (19.97%)	770,112 (12.53%)

### Differential gene expression during early flowering

**Figure 7:**
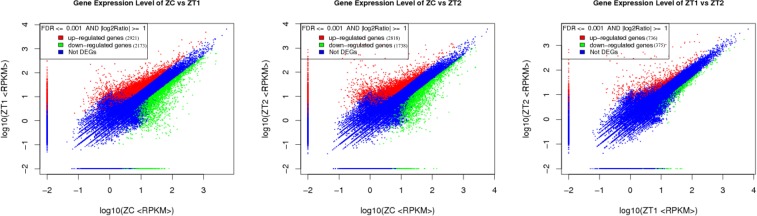
The different expression genes involved in the early flowering of *AS*. Result indicates the number of up- and down-regulated genes between ZC and ZT1, ZC and ZT2, and ZT1 and ZT2.

The gene expression changes in different stages of the early flowering of *AS* were screened by DGE analysis. RPKM was applied to assess the changes in gene expression. There were 5094 genes markedly changed between ZC and ZT1, with 2921 and 2173 of them being up- and down-regulated. Between ZC and ZT2, 4556 DEGs were screened, with 2818 up-regulated and 1738 down-regulated. There were 1111 DEGs markedly changed between ZT1 and ZT2, with 736 and 375 of them being up- and down-regulated. These data are presented in a histogram diagram in Fig. 7.

### Predicted genes involved in early flowering of *AS*

**Figure 8:**
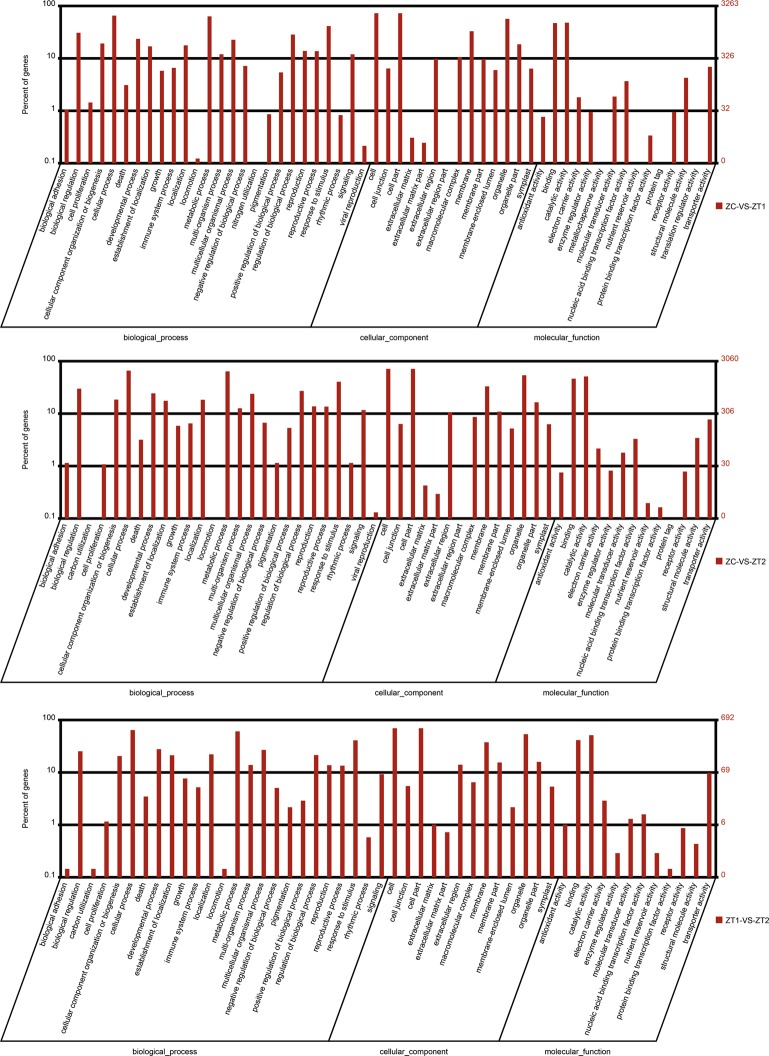
Gene ontology (GO) classification of DEGs between ZC and ZT1, ZC and ZT2, and ZT1 and ZT2. DEGs are annotated in three categories: biological process, cellular component, molecular function. The x-axis indicates the names of each GO term. The right and left y-axis indicate the number and the percentage of each GO term respectively.

In the “cellular component” ontology category, there were 14, 14 and 13 enriched terms in the ZC vs. ZT1, ZC vs. ZT2 and ZT1 vs. ZT2, comparisons, respectively. In the “molecular function” category, there were 15, 13 and 12 enriched terms in the ZC vs. ZT1, ZC vs. ZT2 and ZT1 vs. ZT2, comparisons, respectively. In the “biological process” category, there were 26, 26 and 25 enriched terms in the ZC vs. ZT1, ZC vs. ZT2 and ZT1 vs. ZT2, comparisons, respectively (Fig. 8).

In order to further study the functions of DEGs, pathway enrichment analysis was performed on annotated DEGs. The KEGG pathway was considered significantly enriched with corrected P value < 0.05. The top 10 enriched KEGG pathways related to DEGs observed in different samples of ZC, ZT1 and ZT1 plants were listed in Table S1, Table S2 and Table S3, respectively. The DEGs between ZC and ZT were focused in pathways, such as “Plant hormone signal transduction”, “Biosynthesis of secondary metabolites”, “Plant-pathogen interaction” and so on.

### Key genes involved in flower development

Genes differentially expressed between ZC and ZT1, ZT2 were screened out. Genes having an adjusted log2 ≥ 1 or log2 ≤ −1 found by DGE were assigned as DEGs. There were many genes showing significantly different expression levels (Table 5).

**Table 5:** Statistical results of DEGs annotations.

ID	Gene name		ZT1/ZC		ZT2/ZC	
			Ratio	P value	Ratio	P value
CL5543.Contig2_DGT	The Photoperiodic pathway	PHYOCHROME A(PHYA)	2.02↑	3.17E-05	2.05↑	2.12E-05
CL7271.Contig2_DGT		CONSTANS(CO)	7.96↑	0.120782	8.64↑	0.033365
Unigene41197_DGT		FLOWERING LOCUS T(FT)	8.81↑	0.059875	→	→
CL4589.Contig2_DGT		GIGANTEA(GI)	3.46↑	0.491492	4.41↑	0.258322
CL5603.Contig3_DGT		PHYOCHROME B(PHYB)	−5.34↓	0.508582	0.97↑	0.641554
CL311.Contig3_DGT		EARLY FLOWERING 4(ELF4)	−1.78↓	2.57E-08	−1.65↓	8.86E-08
CL2028.Contig5_DGT	The vernalization pathway	FLOWERING LOCUS C(FLC)	6.14↑	0.243646	→	→
CL2088.Contig1_DGT		FRIGIDA(FRI)	6.72↑	0.491492	8.25↑	0.130579
Unigene3151_DGT		VERNALIZATION 1(VRN1)	9.57↑	3.95E-07	9.76↑	3.96E-08
CL582.Contig3_DGT		VERNALIZATION INSENSITIVE 3(VIN3)	6.42↑	0.029682	6.37↑	0.033365
CL7283.Contig1_DGT		SUPPRESSOR OF OVEREXPRESSION OF CO 1(SOC1)	−7.86↓	4.08E-69	−4.60↓	7.50E-59
CL4574.Contig4_DGT		AGAMOUS	12.38↑	7.98E-71	13.23↑	2.01E-128
Unigene30906_DGT	The autonomous pathway	FCA	−1.33↓	0.00178	0.87↑	0.0577016
Unigene7256_DGT		FPA	→	→	→	→
Unigene13101_DGT		FLOWERING TIME CONTROL PROTEIN(FY)	→	→	→	→
CL1798.Contig6_DGT		FVE	→	→	→	→
CL1178.Contig2_DGT	The gibberellin pathway	gibberellin 3beta-hydroxylase2	9.27↑	0.000218	8.80↑	0.002178
CL9718.Contig2_DGT		gibberellin 20-oxidase	→	→	→	→
Unigene30596_DGT		LEAFY	9.87↑	5.25E-12	8.42↑	7.19E-05
Unigene35571_DGT	Inhibit gene	TFL1	8.55↑	0.059875	→	→
CL8673.Contig1_DGT		CLF	4.26↑	0.243646	→	→

↑: up-regulated, ↓: down-regulated, → : no difference.

In the study of the flowering mechanism of plant, most of the key genes are involved in photoperiodic pathway, vernalization pathway, autonomous pathway, gibberellin pathway. In photoperiodic pathway, we detected an increase in the expression of four genes (*PHYA*, *CO*, *FT* and *GI*) in the early flowering *AS*, the expression of the two genes (PHYB and ELF4) decreased, and the expression of the five genes (*PHYC*, *CRY1*, *CRY2*, *LHY* and *CCA1*) remained unchanged. In vernalization pathway, we detected an increase in the expression of four genes (*FLC*, *FRIGIDA*, *VRN1* and *VIN3*) in the early flowering *AS*, the expression of the *SOC1* decreased. In gibberellin pathway, we detected an increase in the expression of two genes (*GA3ox*, *LFY*) in the early flowering *AS*, and the expression of the *gibberellin 20-oxidase* remained unchanged. In autonomous pathway, all 4 key genes (FCA, FPA, FY and FVE) were no significant difference.

### Gene expression changes analysis by qRT-PCR

**Figure 9:**
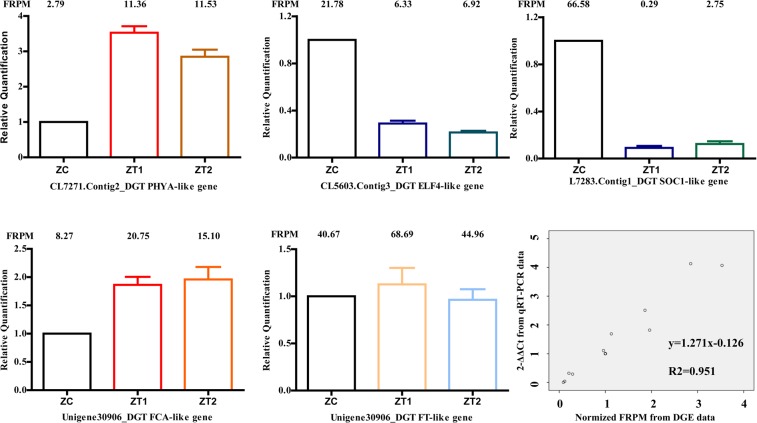
qRT-PCR validation of expression levels of candidate genes from DGE analysis. 9 candidate genes involved in early flowering in *AS* were selected for qRT-PCR to validate the result of DGE data. The x-axis indicates different sample. The y-axis indicates normalized log2 value of gene expression levels. The DGE sequencing data were represented by the FPKM value of different samples.

To confirm the results of DGE, qRT-PCR was applied to analyze the expression of eleven key genes involved in early flowering of *AS*. The expression levels of five genes (*PHYA*, *ELF4*, *SOC1*, *FCA* and *FT*) in ZC, ZT1 and ZT2 were shown in Fig. 9. 2^−ΔΔCt^ method was applied to calculate the relative expression of the genes. The DGE sequencing data was measured by the log2 value of samples. DGE sequencing and qRT-PCR showed significantly positive correlation (R^2^ = 0.951) in linear regression analysis (Fig. 9), suggesting that the result of DGE analysis agreed well with qRT-PCR, thus proved the reliability of sequencing results.

## Discussion

Now, lack of genomic and transcriptome data limited the research of the mechanism of early flowering of *AS*. In the present study, the Illumina sequencing technology were used for *de novo* reference transcriptome assembly using flower buds of early flowering *AS* and apical meristem of vegetative growth *AS*. After RNA sequencing, 68,262 unigenes were assembled. 49,477 (72.5%) unigenes were matched with public databases. Our results will contribute to future genomic studies on *AS* and other Umbelliferae species. However, there were still nearly one third of the unigenes cannot be matched in public databases. Similar phenomena existed in transcriptome assemble of other plant, such as *Lycoris aurea*^11^ and *Tagetes erecta*^12^. The reason may be that the gene expression information of Umbelliferae is too little and the uniqueness of the gene expression of Umbelliferae. DGE was often used in combination with RNA sequencing to screen for differences in gene expression in different tissue of plant or to study disease mechanisms. Thus, a DGE analysis of apical meristem of vegetative growth *AS* and flower buds of early flowering *AS* was carried out to preliminarily clarify the mechanism of early flowering. According to the DGE results, a total of 5,094 and 4,556 transcripts were differently expressed between ZT1 and ZC, as well as ZT2 and ZC.

In Arabidopsis, there are four classic pathways which controlled the flower time. In our study, some key genes in photoperiodic pathway, vernalization pathway and gibberellin pathway are up-regulated in early flowering *AS*. By contrast, all key genes in autonomous pathway are not changed. There are similarities in the gene expression of early flowering in *AS* and normal flowering in model plant, but at the same time there are still some differences in gene expression. These different genes are the focus of our future research work.

In photoperiodic pathway, there were 4 genes (PHYA, CO, FT and GI) expressed higher in ZT. By contrast, there were only two genes (PHYB and ELF4) expressed higher in ZC. There were 5 genes (PHYC, CRY1, CRY2, LHY and CCA1) no significant difference. In phytochromes genes, PHYA was reported can promote flowering. On contrast, PHY, PHYD, PHYE inhibit flowering^13–15^. Our results were agreed with previous findings. In cryptochromes genes, CRY1 and CRY2 were both reported can promote flowering, our results were much the same between other groups^16^. GI and CO are regulated circadian clock, CO was considered as a gene that accelerates flowering in response to long days. FT is the target gene of CO, which is restricted to a similar time of day as expression of CO. FT was considered as one of the three integrons which can promote flowering^17^. CO, FT and GI were all found high expressed in early flowering *AS*. Photoperiodic pathway should be involved in the early flowering phenomenon of *AS*.

In vernalization pathway, there were 4 genes (FLC, FRIGIDA, VRN1 and VIN3) expressed higher in early flowering *AS*. By contrast, there were only one genes (SOC1) expressed higher in ZC. SOC1 is a major floral pathway integrator, which encodes a MADS box transcription factor and is one of the key floral activators integrating multiple floral inductive pathways, namely, long-day, vernalization, autonomous, and gibberellin-dependent pathways^18^, but SOC1 expression is obviously decreased in our experiment. FLC, an upstream negative regulator of SOC1, is high expressed, although VRN1 and VIN3 which control vernalization-mediated FLC silencing are both high expressed^19^. This should the reason of SOC1 expression decreased.

Gibberellins (GAs) are essential for the development of fertile flowers in many plants, and may also be required immediately after fertilization^20,21^. In the GA-biosynthetic pathway, GA 20-oxidases and gibberellin 3 beta-hydroxylase 2 are both key enzymes^22^. In our study, gibberellin 3 beta-hydroxylase 2 were expressed higher in early flowering *AS* and there were no significant difference in gibberellin 20-oxidase expression level. The LFY homologs play a major role in the initiation of flowering^23^. LFY was also considered as one of the three integrons which can promote flowering^24^, which was positive regulated by GA. In our study, LFY was higher expressed in early flowering *AS*. Gibberellin pathway should be involved in the early flowering phenomenon of *AS*.

A central player in the floral transition is the floral repressor FLC^25^, the MADS-box transcriptional regulator that inhibits the activity of genes required to switch the meristem from vegetative to floral development^26,27^. One of the many pathways that regulate FLC expression is the autonomous promotion pathway composed of FCA, FY, FLD, FPA, FVE, LD, and FLK^28^. In our experiment, all 4 key genes (FCA, FPA, FY and FVE) were no significant difference. The proteins involved in autonomous pathway have no changes in early flowering in *AS*.

In fact, in addition to the classic four pathways that regulate plant flowering, we have also discovered changes in the expression of other genes. Plant polyamines are also an important class of plant growth regulators. Arginine decarboxylase (ADC)^29^, S-adenosylmethionine synthetase (SAMS), S-adenosylmethionine decarboxylase (SAMDC)^30^, Spermidine synthase (SPDS)^31^, polyamine oxidase (PAOs) are key enzymes in polyamine metabolism. *ADC*, *SAMDC* and *SPDS* expression are up-regulated in early flowering sample.

In conclusion, early flowering of *AS* was major effected by the genes involved in photoperiodic pathway and GA pathway. Vernalization pathway and autonomous pathway no significantly changes in early flowering. This also should be the difference between the early flowering and normal flowering. These results provide basic information for exploring the molecular mechanisms that influence the early flowering of *AS*.

## Conclusion

Now, effective genetic information on *AS* is very limited. Here, we combined RNA-Seq and DGE to study the molecular mechanism of early flowering of *AS*. We got 49,183,534 clear reads and assembled into 68,262 unigenes, the average length of each unigene was 728 bp.

**Figure 10:**
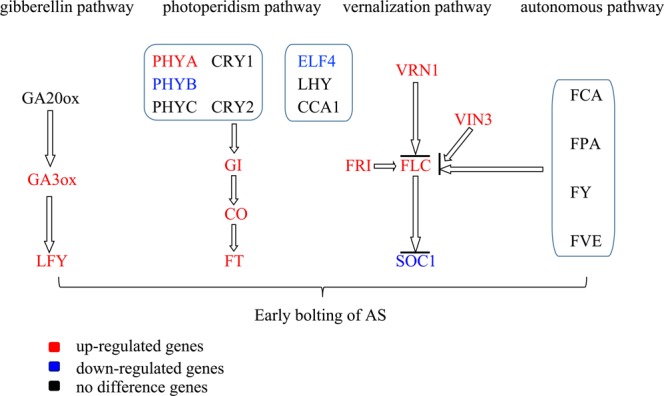
The mechanism of early flowering of *AS*.

The result of sequencing provided effective gene expression profile information for genomic research of *AS*. Based on DGE study, many important genes regulating early flowering of *AS* were discovered and further analyzed. In this paper, we proposed a putative network underlying an overview of known floral regulators present and differentially regulated during floral induction of *AS* (Fig. 10), which provided an important reference for the study of the molecular mechanisms of early flowering in *AS*.

## Supplementary information

Dataset 1
