# Author Correction: Transcriptome and digital gene expression analysis unravels the novel mechanism of early flowering in *Angelica sinensis*

**DOI:** 10.1038/s41598-020-58024-4

**Published:** 2020-01-31

**Authors:** Guang Yu, Yuan Zhou, Juanjuan Yu, Xueqin Hu, Ye Tang, Hui Yan, Jinao Duan

**Affiliations:** 1 School of Medicine and Life Sciences, Nanjing University of Chinese Medicine, Nanjing, China; 2 Jiangsu Collaborative Innovation Center of Chinese Medicinal Resources Industrialization, Nanjing University of Chinese Medicine, Nanjing, China

Correction to: *Scientific Reports* 10.1038/s41598-019-46414-2, published online 11 July 2019

In the original version of this Article, Guang Yu and Yuan Zhou were omitted as equally contributing authors. This has now been corrected in the PDF and HTML versions of the paper.

