# Supplementary material for: Transcriptome and digital gene expression analysis unravels the novel mechanism of early flowering in Angelica sinensis
Source: Sci Rep. 2019 Jul 11;9:10035. doi: 10.1038/s41598-019-46414-2 (PMC6624268; doi:10.1038/s41598-019-46414-2)
Supplement: Supplementary file 1 — Dataset 1 [file 41598_2019_46414_MOESM1_ESM.pdf]

Transcriptome and digital gene expression analysis unravels the novel mechanism of early flowering in Angelica sinensis

Guang Yu 1, 2*, Yuan Zhou 1, Juanjuan Yu 1, Xueqin Hu 1, Ye Tang 1, Hui Yan 2, Jinao Duan 2*

Table S1. The top 10 annotated unigene pathways in ZC-VS-ZT1.

Pathway	DEGs with pathway annotation (2443)	All genes with pathway annotation (26, 438)	Pvalue	Qvalue	Pathway ID
Ribosome	130 (5.32%)	455 (1.72%)	1.106758e-32	1.394515e-30	ko03010
Biosynthesis of secondary metabolites	377 (15.43%)	2807 (10.62%)	8.078185e-15	5.089257e-13	ko01110
Plant hormone signal transduction	216 (8.84%)	1476 (5.58%)	3.808199e-12	1.599444e-10	ko04075
Stilbenoid, diarylheptanoid and gingerol biosynthesis	48 (1.96%)	200 (0.76%)	5.198042e-10	1.637383e-08	ko00945
Cutin, suberine and wax biosynthesis	35 (1.43%)	136 (0.51%)	1.646063e-08	4.148079e-07	ko00073
Endocytosis	97 (3.97%)	614 (2.32%)	1.135344e-07	2.384222e-06	ko04144

Ether lipid metabolism	68 (2.78%)	400 (1.51%)	6.273643e-07	1.129256e-05	ko00565
Limonene and pinene degradation	37 (1.51%)	171 (0.65%)	7.749235e-07	1.220505e-05	ko00903
Flavone and flavonol biosynthesis	25 (1.02%)	101 (0.38%)	3.775099e-06	5.285139e-05	ko00944
Flavonoid biosynthesis	38 (1.56%)	209 (0.79%)	3.989874e-05	5.027241e-04	ko00941

Table S2. The top 10 annotated unigene pathways in ZC-VS-ZT2.

Pathway	DEGs with pathway annotation (2443)	All genes with pathway annotation (26, 438)	Pvalue	Qvalue	Pathway ID
Biosynthesis of secondary metabolites	387 (16.91%)	2807 (10.62%)	6.302656e-22	7.626214e-20	ko01110
Ribosome	104 (4.54%)	455 (1.72%)	1.899931e-20	1.149458e-18	ko03010
Stilbenoid, diarylheptanoid and gingerol biosynthesis	59 (2.58%)	200 (0.76%)	1.469937e-17	5.928746e-16	ko00945
Cutin, suberine and wax biosynthesis	46 (2.01%)	136 (0.51%)	1.579127e-16	4.776859e-15	ko00073
Phenylpropanoid biosynthesis	80 (3.49%)	428 (1.62%)	3.726825e-11	9.018917e-10	ko00940
Limonene and pinene degradation	42 (1.83%)	171 (0.65%)	4.117851e-10	8.304333e-09	ko00903
Plant hormone signal transduction	196 (8.56%)	1476 (5.58%)	6.710945e-10	1.160035e-08	ko04075

Flavonoid biosynthesis	47 (2.05%)	209 (0.79%)	9.242973e-10	1.337450e-08	ko00941
Carotenoid biosynthesis	44 (1.92%)	189 (0.71%)	9.947972e-10	1.337450e-08	ko00906
Metabolic pathways	603 (26.34%)	5812 (21.98%)	1.333618e-07	1.613678e-06	ko01100

Table S3. The top 10 annotated unigene pathways in ZC-VS-ZT2.

Pathway	DEGs with pathway annotation (515)	All genes with pathway annotation (26, 438)	Pvalue	Qvalue	Pathway ID
Cutin, suberine and wax biosynthesis	23(4.47%)	136(0.51%)	2.324002e-15	1.450126e-13	ko00073
Metabolic pathways	191 (37.09%)	5812 (21.98%)	2.736086e-15	1.450126e-13	ko01100
Biosynthesis of secondary metabolites	102 (19.81%)	2807 (10.62%)	3.629129e-10	1.282292e-08	ko01110
Flavonoid biosynthesis	21(4.08%)	209 (0.79%)	9.969012e-10	2.641788e-08	ko00941
Endocytosis	36 (6.99%)	614 (2.32%)	5.529264e-09	1.172204e-07	ko04144
Ether lipid metabolism	27 (5.24%)	400 (1.51%)	2.721665e-08	4.808275e-07	ko00565
Other glycan degradation	17(3.3%)	187 (0.71%)	1.701029e-07	2.575844e-06	ko00511

Photosynthesis-antenna proteins	8 (1.55%)	38(0.14%)	5.744412e-07	7.611346e-06	ko00196
Carotenoid biosynthesis	16 (3.11%)	189 (0.71%)	1.020386e-06	1.201788e-05	ko00906
Stilbenoid, diarylheptanoid and gingerol biosynthesis	16 (3.11%)	200 (0.76%)	2.151856e-06	2.280967e-05	ko00945
